# Cell Membrane Fatty Acid Composition of *Chryseobacterium frigidisoli* PB4^T^, Isolated from Antarctic Glacier Forefield Soils, in Response to Changing Temperature and pH Conditions

**DOI:** 10.3389/fmicb.2017.00677

**Published:** 2017-04-19

**Authors:** Felizitas Bajerski, Dirk Wagner, Kai Mangelsdorf

**Affiliations:** ^1^Alfred Wegener Institute, Helmholtz Centre for Polar and Marine ResearchPotsdam, Germany; ^2^GFZ German Research Centre for Geosciences, Helmholtz Centre Potsdam, Section 5.3 GeomicrobiologyPotsdam, Germany; ^3^GFZ German Research Centre for Geosciences, Helmholtz Centre Potsdam, Section 3.2 Organic GeochemistryPotsdam, Germany

**Keywords:** bacterial cell membrane, fatty acid composition, cold temperature, physiological adaptation, *Flavobacteriaceae*, biogeochemical gradients, permafrost

## Abstract

Microorganisms in Antarctic glacier forefields are directly exposed to the hostile environment of their habitat characterized by extremely low temperatures and changing geochemical conditions. To survive under those stress conditions microorganisms adapt, among others, their cell membrane fatty acid inventory. However, only little is known about the adaptation potential of microorganisms from Antarctic soil environments. In this study, we examined the adaptation of the cell membrane polar lipid fatty acid inventory of *Chryseobacterium frigidisoli* PB4^T^ in response to changing temperature (0°C to 20°C) and pH (5.5 to 8.5) regimes, because this new strain isolated from an Antarctic glacier forefield showed specific adaptation mechanisms during its detailed physiological characterization. *Flavobacteriaceae* including *Chryseobacterium* species occur frequently in extreme habitats such as ice-free oases in Antarctica. *C. frigidisoli* shows a complex restructuring of membrane derived fatty acids in response to different stress levels. Thus, from 20°C to 10°C a change from less *iso*-C_15:0_ to more *iso*-C_17:1ω7_ is observed. Below 10°C temperature adaptation is regulated by a constant increase of *anteiso*-FAs and decrease of *iso*-FAs. An *anteiso-* and bis-unsaturated fatty acid, *anteiso*-heptadeca-9,13-dienoic acid, shows a continuous increase with decreasing cultivation temperatures underlining the particular importance of this fatty acid for temperature adaptation in *C. frigidisoli*. Concerning adaptation to changing pH conditions, most of the dominant fatty acids reveal constant relative proportions around neutral pH (pH 6–8). Strong variations are mainly observed at the pH extremes (pH 5.5 and 8.5). At high pH short chain saturated *iso*- and *anteiso*-FAs increase while longer chain unsaturated *iso*- and *anteiso*-FAs decrease. At low pH the opposite trend is observed. The study shows a complex interplay of different membrane components and provides, therefore, deep insights into adaptation strategies of microorganisms from extreme habitats to changing environmental conditions.

## Introduction

Microorganisms successfully colonize almost all existing ecological niches, including hostile environments such as hot springs, the deep sea, hot or polar deserts (Rothschild and Mancinelli, 2001). The polar regions are microbial-dominated ecosystems and create perfect conditions for extremophiles (Wynn-Williams, 1996). The Antarctic continent is characterized by extreme climatic conditions, limited nutrient availability, high salinity, low temperatures and low water activity (Cannone et al., 2008). Soil microbial communities are able to respond and adapt to severe environmental conditions such as changing temperature and pH regimes (Ganzert et al., 2011; Bakermans et al., 2012; Bajerski and Wagner, 2013). In general, there are several ways of stress response such as the induction of special heat/cold shock proteins, the production and release of protective compatible solutes or an enhanced/reduced metabolism (Georlette et al., 2004). Another crucial adaptation process is the ability to adjust the cell membrane structure, because a fluid cell membrane is essential for microorganisms to maintain the function of important metabolic systems such as the electron transport chain (Denich et al., 2003). Bacterial cell membranes are mainly formed by polar lipid (PL) bilayers (e.g., phospholipids) best described in the “Mosaic Model” by Singer (Singer, 1972). Microorganisms have developed several mechanisms to change their cell membrane composition in order to maintain cell membrane fluidity and functionality in response to shifting environmental conditions, which is known as homeoviscous adaption (Sinensky, 1974). The membrane PL composition can change regarding the polar head groups or the acyl side chains (Russell, 1984; Boggs, 1986). Microbial PL fatty acid side chains are saturated or monounsaturated (rarely polyunsaturated) fatty acids with 12 to 24 carbon atoms and the acyl side chains can contain branches or ring structures such as cyclopropyl, -pentyl, and –hexyl rings. Thus, the phenotypic adaption of the cell membrane structure can be regulated by the degree of unsaturation, the chain length, branching or cyclisation of bacterial membrane fatty acids (Denich et al., 2003). The adaption to low temperature is realized by a higher proportion of unsaturated fatty acids and a shift to more short chain fatty acids (Suutari and Laakso, 1994). Another adaptation process is the change of *iso-* and *anteiso*-branched fatty acids, whereas the proportion of *anteiso*-fatty acids increases with decreasing temperature (Kaneda, 1991). The incorporation of *cis*-unsaturation, shorter-chained fatty acids and fatty acids with branches or cycles reduces the melting temperature of the cell membrane leading to an increased fluidity at low temperature (Russell, 1989; Mangelsdorf et al., 2009).

Several studies indicate different and variable adaptations of the PL inventory in response to changing pH regimes: A decrease of *iso*-C_15:0_ and *iso*-C_16:0_ at increasing pH and no significant effect of the *anteiso*-branched fatty acids was reported (Bååth and Anderson, 2003) as well as an increase of *anteiso-*C_15:0_ and no change of *iso*-C_15:0_ at higher pH (Männistö et al., 2007). Furthermore, the proportion of unsaturated fatty acid may increase with rising pH (Männistö et al., 2007). The incorporation of a cyclopropane ring may enhance the stability of the cell membrane at low pH, low temperature or in slowly growing cells (Russell, 1989; Brown et al., 1997). Since these results show no clear picture additional knowledge on the pH adaption of the microbial cell membrane is needed.

Unsaturation can be induced post-synthesis by desaturase as a rapid answer system. Other adaptions like chain length or branching need de novo synthesis and thereby require bacterial growth under extreme conditions (Russell, 1984). Former studies provide a general idea of the mechanisms involved in bacterial cell membrane adaptation to changing environmental condition (reviewed in Denich et al., 2003), but the structural adaptation of microorganism from extreme habitats such as Antarctic soils still remain poorly understood. Furthermore, microorganisms are very divers in their biochemistry and physiology and the adaptation of the fatty acid composition in response to stress varies between different bacteria (Russell, 1984; Denich et al., 2003). Studying the effects of different environmental parameters on the cell membrane structure could give new insights in the adaptation mechanisms of the microorganism to the extreme environment, because microorganisms can apply several mechanisms to maintain the fluidity and functionality of the cell membrane under different conditions.

In this study, we focus on the temperature and pH adaption of the cell membrane composition of *Chryseobacterium frigidisoli* PB4^T^, a cold-adapted representative of the *Flavobacteriaceae* family in the phylum *Bacteroidetes*, which was isolated from a mineral soil of a glacier forefield of the Larsemann Hills, East Antarctica (Bajerski et al., 2013). *Flavobacteriaceae* are widely distributed in Antarctic habitats (Abell and Bowman, 2005; Cary et al., 2010) and representatives of the genus *Chryseobacterium* occur frequently in Antarctic soils and sediments (Teixeira et al., 2010; Zdanowski et al., 2013; Gugliandolo et al., 2016). In an ecological study of the earlier mentioned glacier forefield *Chryseobacterium* were detected in the culture-independent clone library sequences and several *Chryseobacterium* strains have been isolated, including *C. frigidisoli* PB4^T^ (Bajerski et al., 2013). This new isolate from an ice-free Antarctic oasis showed special adaptation mechanisms during the detailed physiological description, among others a novel fatty acid was discovered and identified as *anteiso*-heptadeca-9,13-dienoic acid, a ω3,7-fatty acid (Mangelsdorf et al., 2017). We investigated its peculiarities for adaptation to low temperatures and pH values, which are relevant properties in Antarctic soils. More precisely, changes in the membrane fatty acid composition of *C. frigidisoli* were analyzed after incubations of the cells between 0°C and 20°C and in a pH range of 5.5 to 8.5.

## Materials and Methods

### Study Site

The Larsemann Hills are an ice-free oasis situated in the Prydz Bay region in East Antarctica (69°30′S, 76°20′E; Stüwe et al., 1989). The study site is characterized by severe climatic conditions with low temperatures between -29°C and 0°C and little precipitation of about 250 mm a^-1^ (Hodgson et al., 2001). Soil formation processes could not be observed and the studied material can be classified as dry and oligotrophic weathering debris. A detailed site description is given in an environmental study dealing with bacterial succession in two glacier forefields of the Larsemann Hills (Bajerski and Wagner, 2013). The studied bacterial strain was isolated from the weathering debris of the glacier forefield called “Black Valley Transect” in 446 m distance from the glacier (S 69°24.315; E 76°20.295; Bajerski et al., 2013). The harvested profile was characterized by a black organic mat mixed with coarse-grained sandy material at the surface and sandy permanently frozen ground in 1–6 cm depth. The soil pH of the study site ranged from acidic (pH 4.9) to alkaline (8.3), but the studied strain originated from a sampling site with almost neutral pH (6.8).

### Bacterial Strains

**FIGURE 1:**
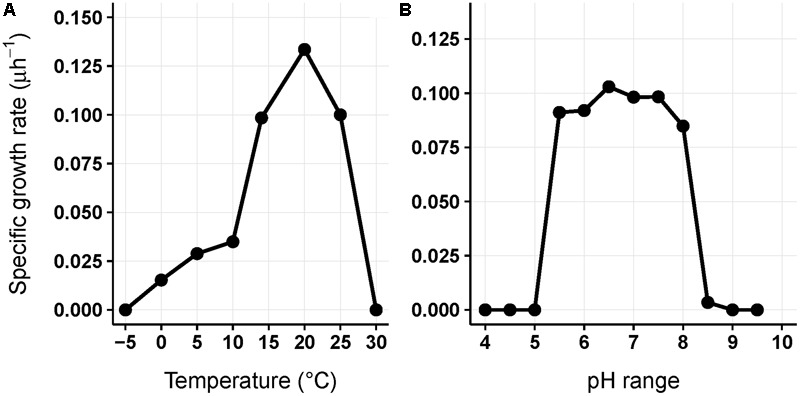
**Physiological characteristics of strain *Chryseobacterium frigidisoli* PB4^T^; specific growth rate in dependence of the temperature (A)** and pH **(B)**, respectively. The specific growth rate μ was determined by measuring turbidity photometrically at 600 nm during incubation in R2A media.

*Chryseobacterium frigidisoli* strain PB4^T^ was chosen to examine the effect of changing temperature and pH on the fatty acid composition of the bacterial cell membrane. This strain was described as a novel psychrotolerant bacterium (Bajerski et al., 2013) being able to grow between 0 and 25°C with an optimum growth at 20°C (**Figure 1A**). The gram-negative bacterium grows at pH values from pH 5.5–8.5 with optimum at pH 6.5 (**Figure 1B**). The turbidity of the cell culture was measured photometrical at 600 nm to determine the optical density (OD) during an incubation in R2A media (0.05% Proteose peptone, 0.05% Casamino acids, 0.05% yeast extract, 0.05% dextrose, 0.05% soluble starch, 0.03% dipotassium phosphate, 0.005% magnesium sulfate 7× H_2_O, 0.03% sodium pyruvate (w/v); pH 7.2; Reasoner and Geldreich, 1985). The specific growth rate μ was determined in the exponential growth phase (linear range) using the measured ODs and was calculated as follows:

$$\mu =\frac{\ln{\text{OD}}_{\text{x}}-\ln{\text{OD}}_{0}}{{\text{t-t}}_{\text{0}}}.$$

To study temperature adaption the strain was incubated at 0, 5, 10, 14, 20°C in R2A medium (pH 7.2). The impact of changing pH values on the membrane composition was analyzed using the R2A medium (Reasoner and Geldreich, 1985) modified with the following buffers: glycine (pH 4.0–5.0 and pH 10.0), MES [2-(*N*-morpholino)ethanesulfonic acid**;** pH 5.0–6.5], HEPES [4-(2-hydroxyethyl)-1-piperazineethanesulfonic acid; pH 7.0-8.0], and BTP ([1,3-bis(tris(hydroxymethyl)methylamino)propane]; pH 8.5–9.5; Hoaki et al., 1994). Growth in dependence of pH was detected at pH 5.5, 6.0, 6.5, 7.0, 7.5, 8.0, and 8.5 after incubation at 18°C (in the range of the temperature optimum). For PL extraction cells from biological triplicates were harvested at the transition from late exponential to early stationary growth phase by centrifuging the combined cultures for 5 min at 10.000 *g*, washing with sterilized tab water and centrifuging again.

### Analytical Procedures

The cell pellets of *C. frigidisoli* cultivated under different temperature and pH conditions were extracted with a flow blending system using a solvent mixture of methanol (MeOH)/dichloromethane (DCM)/ammonium acetate buffer with a ratio of 2:1:0.8 (v/v) for 5 min (modified after Bligh and Dyer, 1959). Afterwards, the solvent ratio of the extract was changed to 1:1:0.9 by adding DCM and ammonium acetate buffer resulting into a phase separation of an organic and an aqueous phase. Subsequently, the aqueous phase was re-extracted three times with DCM. The combined organic phases were concentrated using a Turbo Vap system (Zymark). Afterwards, the extract was chromatographically separated into fractions of different polarity resulting, among other fractions, into a polar lipid (PL) fraction as described in Zink and Mangelsdorf (2004). This standard protocol was used mainly to clean up the PL fraction and to remove free fatty acids from the extract, thus they cannot mix up with the side chain fatty acids from the polar membrane lipids. Here we focused on the PL fraction since it contains the main cell membrane lipids of *C. frigidisoli*. An aliquot of the PL fraction was used for *trans*-esterification to obtain the methylated membrane fatty acids by following the method described by Müller et al. (1990). The aliquot of the PL fraction was dissolved in 50 μl of DCM/MeOH (9:1 v/v) in a 2 ml vial. Afterward 50 μl of trimethylsulfoniumhydroxid (TMSH) was added and the vial was sealed before placing in an oven for 2 h at 70°C. The *trans*-esterified (methylated) fatty acids were dried and subsequently dissolved in 50 μl DCM before analysis on a gas chromatographic system (Trace GC Ultra, Thermo Electron) coupled to a DSQ Thermo Electron Quadrupole mass spectrometer. The GC was equipped with a cold injection system operating in the splitless mode and a SGE BPX 5 fused silica capillary column (50 m length, 0.22 mm ID, 0.25 μm film thickness) using the following temperature program: initial temperature 50°C, 1 min isotherm, heating rate 3°C/min to 310°C, held isothermal for 30 min. Helium was used as a carrier gas with a constant flow rate of 1 mL min^-1^. The injector temperature was programmed from 50 to 300°C at a rate of 10°C s^-1^. Full scan mass spectra were recorded from *m*/*z* 50 to 600 at a scan rate of 2.5 scans s^-1^.

### Statistics

Correlation and significance of the derived data was calculated with IBM SPSS Statistics 21 analyzing bivariate correlation using the Pearson correlation coefficient and testing two-tailed significance.

## Results and Discussion

### Fatty Acid Inventory of *C. frigidisoli* PB4^T^

**FIGURE 2:**
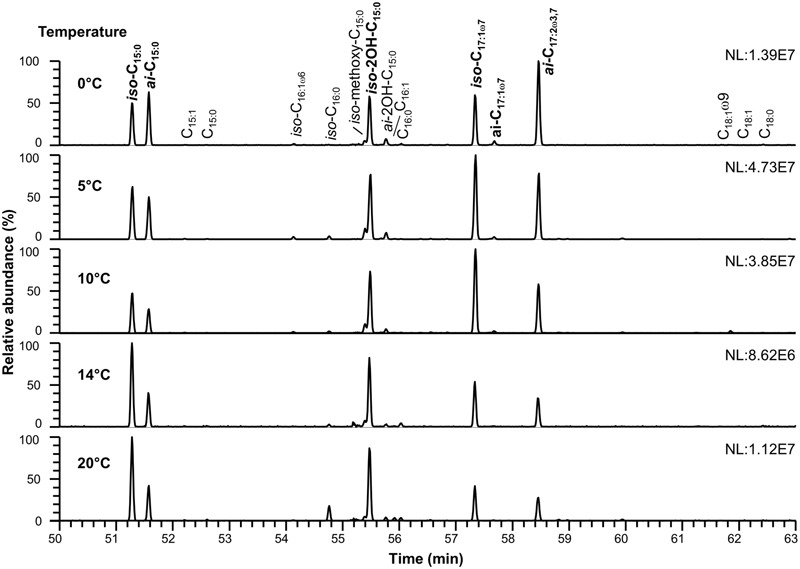
**Cell membrane derived fatty acid inventory of *C. frigidisoli* PB4^T^ at different temperatures.** To study temperature adaption the strain was incubated at 0, 5, 10, 14, 20°C in R2A medium (pH 7.2). The major fatty acids (>10%) are shown in bold. For a detailed list of all membrane fatty acids see **Table 1**.

**FIGURE 3:**
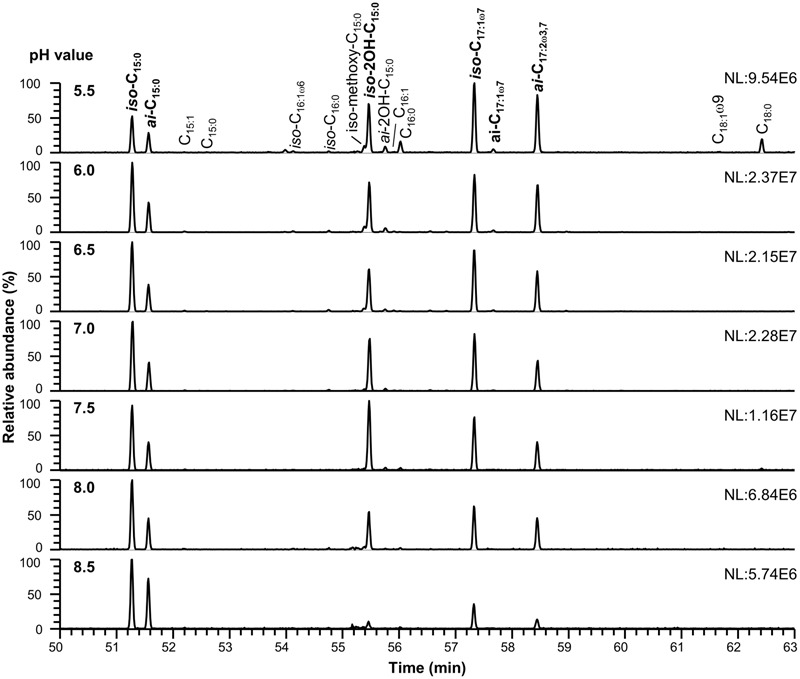
**Cell membrane derived fatty acid of *C. frigidisoli* PB4^T^ at different pH conditions.** Growth in dependence of pH was detected at pH 5.5, 6.0, 6.5, 7.0, 7.5, 8.0, and 8.5 after incubation in R2A medium at 18°C (in the range of the temperature optimum). The major fatty acids (>10%) are shown in bold. For a detailed list of all membrane fatty acids see **Table 2**.

**FIGURE 4:**
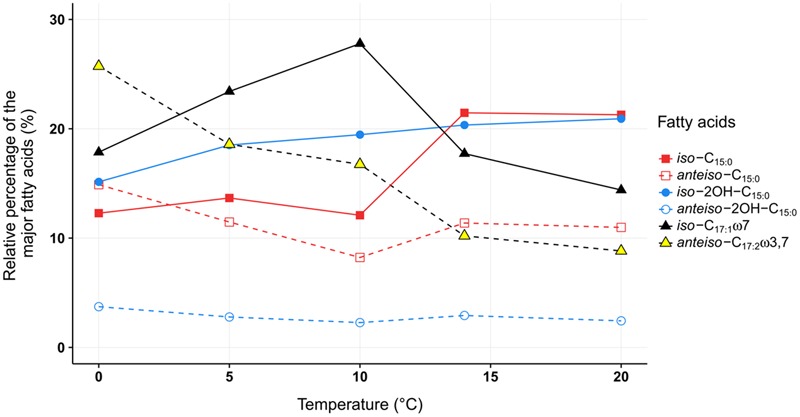
**The relative proportion of the major membrane fatty acids from *C. frigidisoli* PB4^T^ cultivated at different temperatures.** While between 20°C and 14°C only minor variations within the relative proportions of the individual fatty acids were shown, a significant shift from saturated *iso*-C_15:0_ to unsaturated *iso*-C_17:1ω7_ between 14°C and 10°C was observed and below 10°C all *iso*-fatty acids decrease while the *anteiso*-fatty acids are increasing. *iso* = methyl branch at ω-2. *anteiso* = methyl branch at ω-3. C*_x_*_:_*_y_* = *x* number of carbon atoms and *y* number of double bonds. OH = hydroxy group.

The detected polar membrane lipids of *C. frigidisoli* are phosphatidylethanolamines, ornithine lipids as well as flavolipins and flavocristamides. The major fatty acids (>10%, **Figures 2, 3** and **Tables 1, 2**) determined in this study obtained after saponification of membrane lipids are *iso*- and *anteiso*-pentadecanoic acid (*iso-, anteiso*-C_15:0_), *iso*-2-hydroxypentadecanoic acid (*iso*-2OH-C_15:0_), *iso*-heptadeca-9-enoic acid (*iso*-C_17:1ω7_) and a novel fatty acid, which, to our knowledge, has not been previously described. The structure of the newly discovered fatty acid was identified as *anteiso*-heptadeca-9,13-dienoic acid, a ω3,7-fatty acid with a branching position directly at the ω3 double bond. The experiments, which were performed to identify the detailed structure of the newly discovered fatty acid, are described elsewhere (Mangelsdorf et al., 2017). The identified major fatty acids are in accordance with the fatty acid composition of the strain description that identified *iso*-2OH-C_15:0_ and *iso*-C_15:0_ as the major fatty acids (Bajerski et al., 2013). Minor variations between this study and the presented results can be explained by different harvesting time points. In the strain description of Bajerski et al. (2013) cells were harvested at the end of the exponential growth phase, whereas in this study cell material was collected at the transition from late exponential to early stationary growth phase. The predominance of *iso*-2OH-C_15:0_ and *iso*-C_15:0_ at optimum growth seem to be shifted to other C_15_ and longer unsaturated C_17_ fatty acids under sub-optimal conditions (stress/stationary phase). While *iso*-C_15:0_ is predominant in many *Chryseobacterium* species (Vandamme et al., 1994), the high amount of *iso*-2OH-C_15:0_ appears to be distinctive in *C. frigidisoli*. It should be noted that the *iso*-C_17:1ω7_ fatty acid was wrongly labeled as *iso*-C_17:1ω8_ in Bajerski et al. (2013). Other fatty acids such as *iso*-tridecanoic acid (*iso*-C_13:0_), *iso*-tetradecanoic acid (*iso*-C_14:0_), pentadecanoic acid (C_15:0_), *iso*-hexadecenoic acid (*iso*-C_16:1_, presumably *iso*-C_16:1ω6_), *iso*-hexadecanoic acid (*iso*-C_16:0_), *iso*-2-methoxy-pentadecanoic acid (*iso*-2methoxy-C_15:0_), *anteiso*-2-hydroxy-pentadecanoic acid (*anteiso-*2OH-C_15:0_), hexadecanoic acid (C_16:0_), *anteiso*-heptadec-9-enoic acid (*anteiso-*C_17:1ω7_), octadec-9-enoic acid (C_18:1ω9_) and octadecanoic acid (C_18:0_) occur only in minor to trace amounts accounting in total for less than 10 % of the total cell membrane derived fatty acids pattern (**Tables 1, 2**). In some of the minor unsaturated components the exact double bond position could not be assigned to a standard or identified by derivatization experiments (Mangelsdorf et al., 2017), thus, they are simply labeled as unsaturated fatty acids (**Figures 3, 4** and **Tables 1, 2**, respectively).

**Table 1:** Adaption of the cell membrane derived fatty acid inventory of *Chryseobacterium frigidisoli* PB4^T^ with respect to different temperature conditions.

Fatty acid (FA) composition at different temperatures					
(% of total FA)	0°C	5°C	10°C	14°C	20°C
*iso*-C_13:0_	nd	0.30	0.18	0.32	0.48
*iso*-C_14:0_	nd	0.27	0.29	0.65	1.18
C_14:0_	0.29	0.12	0.19	0.46	0.60
***iso*-C_15:0_**	**12.28**	**13.67**	**12.09**	**21.46**	**21.28**
***ai*-C_15:0_**	**14.89**	**11.47**	**8.23**	**11.38**	**10.98**
ratio: *iso/ai*-C_15:0_	0.82	1.19	1.47	1.89	1.94
C_15:1_^∗^	0.30	0.42	0.38	0.68	0.66
C_15:0_^∗^	0.19	0.35	0.25	0.96	1.07
*iso*-C_16:1ω6_	1.26	1.47	1.08	0.61	0.85
*iso*-C_16:0_	0.37	1.24	1.23	1.60	5.78
*iso*-2methoxy-C_15:0_	2.27	3.05	3.20	2.40	1.39
***iso*-2OH-C_15:0_^∗^**	**15.14**	**18.51**	**19.46**	**20.34**	**20.92**
*anteiso*-2OH-C_15:0_	3.73	2.78	2.27	2.92	2.43
ratio: *iso/ai*-OH-C_15:0_	4.06	6.65	8.58	6.97	8.60
C_16:1_	nd	0.42	0.40	1.48	1.89
C_16:0_	1.19	0.36	0.50	2.95	1.88
sum short chain FA	56.79	62.27	59.80	77.07	81.93
***iso*-C_17:1ω7_**	**17.86**	**23.41**	**27.79**	**17.73**	**14.40**
*br*-C_17:1_	0.50	0.39	0.44	nd	nd
*anteiso*-C_17:1ω7_^∗^	2.68	1.44	1.60	1.16	0.91
***anteiso*-C_17:2_**_ω_**_3,7_^∗∗^**	**25.73**	**18.58**	**16.75**	**10.20**	**8.82**
*iso*-2OH-C_16:0_	nd	0.47	0.62	0.39	1.11
C_17:1_	nd	0.26	0.26	0.32	0.58
2OH-C_16:1_	0.40	0.75	0.70	0.49	1.27
C_18:1ω9_	0.29	0.09	0.13	0.58	0.72
C_18:1ω7_	nd	nd	1.70	nd	nd
C_18:0_	0.64	0.19	0.26	0.92	0.79
Sum long chain FA	48.10	45.58	50.25	31.79	28.60
Ratio: short/ long chain FA	1.18	1.37	1.19	2.42	2.86

*To study temperature adaption the strain was incubated at 0, 5, 10, 14, 20°C in R2A medium (pH 7.2). br = branched fatty acid with unknown branching position. ^∗^The correlation of temperature and fatty acid using Pearson correlation coefficient testing two-tailed significance is significant at the 0.05 level (2-tailed). ^∗∗^Correlation is significant at the 0.01 level (2-tailed). nd = not detected. *iso* = methyl branch at ω-2. *anteiso* (*ai*) = methyl branch at ω-3. C_*x:y*_ = x number of carbon atoms and y number of double bonds. OH = hydroxy group. The major fatty acids are shown in bold.*

**Table 2:** Adaption of the cell membrane derived fatty acid inventory of *C. frigidisoli* PB4^T^ to different pH conditions.

Fatty acid (FA) composition at different							
pH (% of total FA)	pH 5.5	pH 6	pH 6.5	pH 7	pH 7.5	pH 8	pH 8.5
*iso*-C_13:0_^∗^	0.29	0.21	0.21	0.21	0.28	0.62	0.97
*iso*-C_14:0_	0.24	0.33	0.46	0.38	nd	0.30	0.78
C_14:0_	0.51	0.15	0.12	0.10	0.27	0.37	0.34
***iso*-C_15:0_^∗^**	**11.07**	**21.32**	**23.12**	**23.43**	**20.76**	**23.38**	**31.01**
***anteiso*-C_15:0_^∗^**	**6.78**	**10.72**	**10.18**	**10.95**	**10.41**	**12.67**	**24.08**
ratio: *iso/ai*-C_15:0_	1.63	1.99	2.27	2.14	1.99	1.85	1.29
C_15:1_^∗^	0.31	0.57	0.71	0.66	0.55	0.69	0.85
C_15:0_^∗^	0.60	0.31	0.30	0.37	0.26	0.25	nd
*iso*-C_16:1ω6_	1.28	0.90	0.68	0.56	0.54	1.20	1.24
iso-C_16:0_	0.86	1.14	1.21	0.97	0.37	1.19	1.16
*iso*-2methoxy-C_15:0_	2.40	2.18	1.33	1.19	0.88	1.85	2.76
***iso*-2OH-C_15:0_**	**15.68**	**17.16**	**16.58**	**19.29**	**24.34**	**15.97**	**6.57**
*anteiso*-2OH-C_15:0_^∗^	3.05	2.61	1.70	1.96	2.30	1.73	nd
ratio: *iso/ai*-2OH-C_15:0_	5.14	6.57	9.74	9.86	10.59	9.24	nd
C_16:1_	nd	0.71	0.76	0.70	nd	nd	nd
C_16:0_	4.39	0.41	0.36	0.59	1.53	1.81	1.29
sum short chain FA^∗^	54,23	67,28	69,73	73,36	75,07	73,12	72,34
***iso*-C_17:1ω7_**	**23.21**	**20.85**	**24.04**	**22.94**	**21.96**	**21.11**	**18.20**
*br*-C_17:1_	0.89	0.31	0.44	0.47	0.57	0.57	1.31
*anteiso*-C_17:1_	2.35	1.53	1.05	0.89	0.83	0.55	1.26
***anteiso*-C_17:2ω3,7_^∗∗^**	**19.03**	**17.33**	**15.46**	**12.85**	**11.88**	**13.62**	**6.41**
*iso*-2OH-C_16:0_	0.57	0.26	0.21	0.26	0.22	0.50	nd
C_17:1_^∗^	0.28	0.42	0.48	0.41	0.15	nd	nd
2OH-C_16:1_	0.37	0.27	0.24	0.35	0.25	0.47	nd
C_18:1ω9_	0.98	0.12	0.14	0.20	0.41	0.46	0.89
C_18:0_	4.89	0.19	0.20	0.27	1.26	0.70	0.88
Sum long chain FA^∗^	52,57	41,28	42,26	38,64	37,53	37,98	28,95
Ratio: short/ long chain FA	1,03	1,63	1,65	1,90	2,00	1,93	2,50

*Growth in dependence of pH was detected at pH 5.5, 6.0, 6.5, 7.0, 7.5, 8.0, and 8.5 after incubation in R2A media at 18°C (in the range of the temperature optimum). br = branched fatty acid with unknown branching position. ^∗^The correlation of pH and fatty acid using Pearson correlation coefficient testing two-tailed significance is significant at the 0.05 level (2-tailed). ^∗∗^Correlation is significant at the 0.01 level (2-tailed). nd = not detected. *iso* = methyl branch at ω-2. *anteiso* (*ai*) = methyl branch at ω-3. C_*x:y*_ = x number of carbon atoms and y number of double bonds. OH = hydroxy group. The major fatty acids are shown in bold.*

Overall, the fatty acid inventory of *C. frigidisoli* is characterized by branched (*iso/anteiso*) and unsaturated fatty acids in the shorter chain range between 15 and 17 carbon atoms. These features are used for instance by cells that are able to grow under harsh living conditions such as low temperatures or changing biogeochemical gradients to maintain membrane fluidity and functionality (Denich et al., 2003). By those means *C. frigidisoli* is well adapted to a psychrotolerant life style, because shorter chain fatty acids, branches and unsaturations increase the fluidity of the cell membrane at low temperatures (Russell, 1984). Furthermore, the whole genus *Chryseobacterium* is characterized by the presence of branched C_15_ and C_17_ fatty acids (Vandamme et al., 1994). The incorporation of *anteiso*-fatty acids, as it is shown in this study or for other psychrotolerant species such as *C. frigidum* (Kim et al., 2016) and *C. haifense* (Hantsis-Zacharov and Halpern, 2007) lowers the melting temperature of the cell membrane enhancing motion ability. *Anteiso*-C_15:0_ (25.5°C) for instance has a critical lower main phase transition temperature than *iso*-C_15:0_ (52.2°C; Suutari and Laakso, 1994). The incorporation of an unsaturation leads to an even stronger decrease in the melting temperature, while for instance a C_16:0_ fatty acid has a solid-liquid phase transition temperature of 63°C, whereas C_16:1ω7_ has a melting temperature of –1°C (Knothe and Dunn, 2009). In line with this, *C. antarcticum* incorporates *iso*-C_15:1_ as the major fatty acid and *C. frigidisoli* and its closest relatives *C. humi* (Pires et al., 2010) and *C. marinum* (Lee et al., 2007) contain various unsaturated C_17_ fatty acids in their cell membranes. In contrast, saturated and longer chain fatty acids (>C_17_), representing adaptation to warmer conditions, play only a minor role in the fatty acid inventory of *C. frigidisoli*. In general, the genus *Chryseobacterium* comprises several psychrotolerant species isolated from cold-affected habitats indicating that *Chryseobacterium* have developed suitable genetic, biochemical, and physiological adaptations for a life at low temperature (Yi et al., 2005; Zhao et al., 2011; Bajerski et al., 2013).

### Fatty Acid Adaption to Variable Temperatures

As discussed in the previous paragraph the cell membrane of *C. frigidisoli* predominately contains branched shorter chained fatty acids partly with unsaturation as an adaptation to a psychrotolerant lifestyle. In the current study, we conducted temperature cultivation experiments at 0, 5, 10, 14, and 20°C. Between 20°C and 14°C the relative proportions of the fatty acids are not much different, however, below 14°C drastic changes are visible (**Figure 4**). This pattern is in accordance with the growth rate curve (**Figure 1A**) showing a significant change in the growth rate at 10°C. Generally, the fatty acid inventories at different cultivation temperatures are composed of the same fatty acids (**Figure 2**). However, there are differences in the relative distributions of the membrane fatty acids. While the *anteiso*-C_15:0_ is more abundant than the *iso*-congener in the 0°C culture, the ratio of *iso-* to *anteiso-*C_15:0_ changes drastically with increasing cultivation temperature with a clear dominance of the *iso*-C_15:0_ in the 20°C culture (**Figure 4**). This change is clearly resembled in the *iso/anteiso*-C_15:0_ ratio in **Table 1**. The relative proportion of *iso*-2OH-C_15:0_ constantly increases from the 0°C to the 20°C culture (**Figure 4**), while the *anteiso*-2OH-C_15:0_ shows an overall slight decrease between 0°C and 10°C and a more or less constant trend between 10°C and 20°C (**Figure 4**). The ratio of *iso/anteiso*-2OH-C_15:0_ (**Table 1**) shows overall an increasing trend from 0°C to 20°C, however, the ratio value of the 10°C culture falls out of this trend due to the lower amount of *anteiso*-2OH-C_15:0_. The change of the *iso/anteiso-*ratio allows the organism to adapt the cell membrane melting temperature in response to different ambient temperatures. The importance of *anteiso*-branched fatty acids at low temperatures was also reported in the temperature adaptation of *Bacillus subtilis* by Klein et al. (1999). They observed a relative dominance of *anteiso-*C_15:0_ and *anteiso-*C_17:0_ in the fatty acid inventory in cold shock experiments with *B. subtilis*.

Another adaptation to ambient temperature can be the chain length of the membrane fatty acids, which can be reduced with decreasing temperatures, since the melting temperature of the respective membrane fatty acids decreases with shorter chain length (Russell, 1989; Suutari and Laakso, 1994). However, in our study on *C. frigidisoli* the chain length does not seem to be a major regulation mechanism (**Table 1**) in response to changing temperature regimes. The overall chain length (short vs. long chain length) does not change significantly with temperature. Nevertheless, the fatty acid inventory of *C. frigidisoli* generally contains a high amount of shorter-chained fatty acids and the species uses other mechanisms (*iso/ai* ratios, unsaturation) to maintain the cell membrane fluidity at low temperature. In contrast to the chain length, temperature adaptation via the relative abundance of unsaturated fatty acids seems to play a significant role. The highly abundant monounsaturated *iso*-C_17:1ω7_ fatty acid shows a strong increase from 20°C to 10°C, which can be interpreted as a temperature adaptation, since a higher relative proportion of unsaturated fatty acids lowers the membrane solid-liquid phase transition temperature (Russell, 1989), in strain PB4^T^ leading to an increased fluidity of the cell membrane (**Figures 2, 4**). At the same time the *iso*-C_15:0_ strongly decreases. Thus, between 20 and 10°C there is a trend from *iso*-C_15:0_ to *iso*-C_17:1ω7_ (**Figure 4**). The *iso*-C_17:1ω7_ has indeed a longer chain length (increase of melting temperature) but the unsaturation (strong decrease in melting temperature) more than compensates the chain length effect and seems to be an adaptation towards lower ambient temperatures. Unsaturation of fatty acids is a very effective temperature adaptation, because it has a higher effect on lipid fluidity and can be either realized by de novo synthesis or modification (desaturation) of already existing fatty acids (Russell, 1984). The longer chain length of *iso*-C_17:1ω7_ might attenuate the adaptation effect introduced by the unsaturation. Below 10°C the relative amount of *iso*-C_17:1ω7_ decreases again. The same is observed for other *iso*-fatty acids. In contrast, the *anteiso*-fatty acids generally increase between 10°C and 0°C (**Figure 4**). Therefore, below 10°C the data indicate that there is an interchange from *iso*-fatty acids to more *anteiso*-fatty acids, which represents a clear adaptation to cooler conditions (Kaneda, 1991). This interchange point in the fatty acid composition is also shown in the growth curve (**Figure 1A**) indicating that *C. frigidisoli* experiences stress at temperatures ≤10°C. Additionally, the *anteiso*-C_17:2ω3,7_ continuously increases (**Figures 2, 4**) from 20°C to 0°C indicating a strong temperature adaptation of *C. frigidisoli* to lower ambient temperatures. The statistical analysis shows that this *anteiso* and bis-unsaturated fatty acid strongly correlates with temperature (*p* < 0.01, *R*^2^ = 0.93). Its relative proportion increases with decreasing temperature and accounts for the highest amount of all fatty acids (25.73%) at 0°C (**Table 1** and **Figures 2, 4**). Thus, the *anteiso-*C_17:3ω3,7_ fatty acid seems to be an important and efficient regulation component for temperature adaption in *C. frigidisoli*. Detailed studies on changes in the PFLA profiles of microorganisms especially from extreme habitats are still rare and the discovery of the new fatty acid shows that the microorganisms develop various ways of stress response. There are several studies reporting the critical role of reducing the fatty acid chain length and altering the branching from *iso* to *anteiso* in reaction to low ambient temperatures (Paton et al., 1978; Annous et al., 1997). Russell (1984) stated that unsaturation is more effective than chain length reduction in low temperature adaptation. However, Mangelsdorf et al. (2009) showed that in microbial communities from Siberian permafrost the temperature regulation was mainly realized by chain length and the trend in the relative proportion of saturated and unsaturated fatty acids did not change significantly. Chain length and desaturation played also a minor role in the cold shock response of *Bacillus subtilis* and evidence for the dominance of *anteiso* branched fatty acids in the adaptation to low temperature was given (Klein et al., 1999). In our study, we observe a high importance of *iso-* and *anteiso-*branched fatty acids in cold adaption. Unsaturated fatty acids such as the *iso*-C_17:1ω7_ show a concerted interplay between these structural species at different temperatures most likely induced by different temperature stress levels. Additionally, the newly discovered bis-unsaturated fatty acid *anteiso*-heptadeca-9,13-dienoic acid plays a major role in the cell membrane temperature adaption of *C. frigidisoli*.

### Fatty Acid Adaption to Shifting pH Values

**FIGURE 5:**
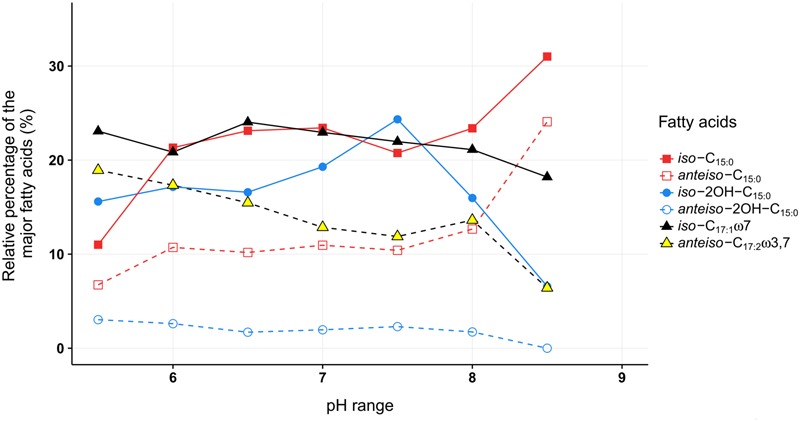
**The relative proportions of the major fatty acids from *C. frigidisoli* PB4^T^ cultivated at different pH conditions.** The pH adaption is characterized by a relatively uniform fatty acid composition at neutral pH and significant variations mainly at the pH extremes. *iso* = methyl branch at ω-2. *anteiso* = methyl branch at ω-3. C*_x_*_:_*_y_* = *x* number of carbon atoms and *y* number of double bonds. OH = hydroxy group.

For most of the major fatty acids (*iso*-C_17:1ω7_, *iso*-C_15:0_, *anteiso-*C_15:0_, *anteiso-*2OH-C_15:0_) of *C. frigidisoli* the relative abundance is rather constant at neutral pH with a plateau phase between pH 6.0 and 8 (**Figure 5**). An exception forms *iso*-2OH-C_15:0_, which shows a maximum at pH 7.5. At the same time membrane fatty acids at pH minima and maxima (**Figure 1B**) show different compositions and variable adaptation mechanisms (**Figure 5**). At pH 8.5 the saturated *iso*- and *anteiso-*C_15:0_ show a significant increase, while the unsaturated *iso*-C_17:1ω7_, *anteiso*-C_17:3ω3,7_ as well as the hydroxy fatty acids *iso*-2OH-C_15:0_ and *anteiso-*2OH-C_15:0_ are all decreasing. In contrast, at pH 5.5 *iso*-C_17:1ω7_, *anteiso-*C_17:3ω3,7_ and *anteiso-*2OH-C_15:0_ increase, while *iso*- and *anteiso-*C_15:0_ decrease. The *iso*-2OH-C_15:0_ also decrease at lower pH. Thus, some opposite compositional trends can be observed at the different pH extremes outlined by the growth range of *C. frigidisoli* (**Figure 1B**).

Overall, the ratio of *iso*- to *anteiso*-C_15:0_ is high at neutral pH (dominance of *iso*-congener) and regardless to the increasing or decreasing trend of the *iso*- and *anteiso*-congeners the ratio becomes lower towards the pH extremes indicating an increasing relative importance of the *anteiso-*branched fatty acids in response to high and low pH (**Table 2**). The introduction of more *anteiso*-branched fatty acids supports a higher flexibility of the cell membrane (Russell, 1989). The main challenge for the microorganism in an acidic or alkaline environment is the change of the ion strength that influences proton motive force of the cell membrane. The maintenance of the transmembrane electrical potential (Δψ) and the transmembrane ΔpH is mainly realized using transporters including active proton transport (Krulwich et al., 2011). A higher flexibility of the cell membrane could enable the incorporation of new membrane channels generating the ion transport through the cell membrane in reaction to the changing ion strength at different pH values.

Furthermore, different branching patterns in response to changing pH were discussed in literature before, which partly disagree with the results of the current study. Bååth and Anderson (2003) reported a decrease of *iso*-C_15:0_ and *iso*-C_16:0_ at increasing pH and no significant effect of the *anteiso*-branched fatty acids, while Männistö et al. (2007) observed an increase of *anteiso-*C_15:0_ and no change of *iso*-C_15:0_ at higher pH in bacterial communities in Arctic Fjelds of Finnish Lapland. Furthermore, an increase of branched (*iso, anteiso*) fatty acids was observed in response to low pH (Russell, 1984). In our study the relative proportions of the *iso*- and *anteiso-*C_15:0_ increases at high pH and, interestingly, those of *iso*- and *anteiso-*2OH-C_15:0_ decrease (**Table 2** and **Figures 3, 5**). Thus, the hydroxy group as the only structural difference among these branched C_15:0_ fatty acids seems to be less favorable for adaptation towards higher pH conditions. In contrast at pH 5.5 the *iso*-C_15:0_ and *anteiso-*C_15:0_ fatty acids significantly decrease, while the hydroxy congeners only slightly decrease (*iso*) or increase (*anteiso*). To our knowledge, this is the first study showing a pH dependent change in the relative amount of methyl and hydroxy-side chains of the fatty acids and we can only assume that an additional hydroxy group is somehow less favorable at alkaline pH in contrast to a lower pH. For acidophilic archaea it was shown that hydroxy groups on sugar moieties prevent the protons from penetrating the cell membrane at low pH (Wang et al., 2012). This shows that a hydroxylation might be a suitable adaptation mechanism in an acidic environment, which could explain why the relative proportion of the *iso*- and *anteiso-*2OH-C_15:0_ is only less affected at lower pH.

Another interesting observation concerns the chain length of the respective fatty acids. *C. frigidisoli* incorporates significantly more short chain fatty acids, predominantly C_15_, at higher pH (**Figures 3, 5**). The unsaturated *iso*-C_17:1ω7_ and the *anteiso*-C_17:2ω3,7_ significantly decrease at higher pH (**Table 2** and **Figure 2**). At low pH the opposite trend can be observed, while *iso*-C_15:0_, *anteiso*-C_15:0_, *iso*-2OH-C_15:0_ all decrease (*anteiso*-2OH-C_15:0_ stays rather constant), *iso*-C_17:1ω7_ and *anteiso*-C_17:2ω3,7_ increase (**Figure 5**). A similar shift from short-chained saturated (pH 7) to long-chained mono-unsaturated fatty acids with decreasing pH was observed in *Streptococcus mutans* and other oral bacteria as a survival strategy in acidic environment, however, the function of this shift is currently not understood (Quivey et al., 2000; Fozo et al., 2004).

Similar to the temperature cultivation experiments, the *anteiso*-C_17:2ω3,7_ fatty acid also seems to play a role in the cell membrane adaption at different pH conditions. With exception of the culture from pH 8, this fatty acid constantly decreases with increasing pH.

In contrast to the temperature adaptation, interpretation of the adaptation mechanisms concerning changing pH condition is rather difficult; in particular, since literature data are somehow contradicting and show no similar trends. Thus, the function of the observed fatty acid variations in the current study is not clear yet. At high pH the decrease in the long chain unsaturated fatty acids seem to support a compaction/stabilization of the cell membrane whereas the concomitantly shift to more short chain *iso*- and *anteiso*-C_15:0_ with a relative increase of the *anteiso*-congener seem to attenuate this effect. At low pH the opposite trend may increase the membrane fluidity. Thus, the pH adaptation of the cell membrane derived fatty acid inventory appears to be a balanced interplay between stabilization and enhanced flexibility of the cell membrane presumably to either prevent or enable protons or other homeostatically active substances passing the cell membrane. Further studies are needed to discover the interrelation between the structural membrane adaptation and the effect on the membrane function at changing pH conditions.

## Conclusion

In its natural Antarctic habitat *C. frigidisoli* PB4^T^ is exposed to extreme low temperatures and changing geochemical gradients. Temperatures fluctuate from summer to winter and especially during diurnal and nocturnal shifts. Geochemical parameters are shaped by a slow succession and severe climatic conditions such as strong winds.

The adaptation of *C. frigidisoli* to low temperatures shows a complex interplay of membrane derived fatty acid variations depending on different stress levels. While between 20°C and 14°C only minor variations within the relative proportions of the individual fatty acids was shown, a significant shift from saturated *iso*-C_15:0_ to unsaturated *iso*-C_17:1ω7_ between 14°C and 10°C was observed and below 10°C all *iso*-fatty acids decrease while the *anteiso*-fatty acids are increasing. All changes in the cell membrane fatty acid inventory represent a clear adaptation to highly fluctuating temperature conditions in the Antarctic environment. Another important finding is the specific role of the recently identified *anteiso*-heptadec-9,13-enoic fatty acid for the temperature adaptation in *C. frigidisoli*, since this fatty acid continuously increases with decreasing temperature and is the dominant fatty acid at 0°C.

The pH adaption is characterized by a relatively uniform fatty acid composition at neutral pH and significant variations mainly at the pH extremes. At high pH a shift from the unsaturated long chain *iso*- and *anteiso*-fatty acids to the saturated short chain *iso*- and *anteiso*-fatty acids is observed while at low pH the opposite trends are visible. These structural differences point to a strengthening of the cell membrane at high pH and a higher fluidity at low pH presumably to enable or prevent exchange processes with the surrounding environment.

Among the importance of the newly identified fatty acid for the temperature adaptation and survival of *C. frigidisoli* in the extreme Antarctic environment, this fatty acid is suggested to serve as a biomarker for the genus *Chryseobacterium* (Mangelsdorf et al., 2017).

## Author Contributions

DW and FB designed the study. FB performed a part of the lab work (microbial physiology) and mainly analyzed and interpreted the data. KM performed the fatty acid analyses of the strain. DW and KM substantial contributed to the interpretation of the results and valuable discussion. FB was drafting the work and DW and KM revised it critically for important intellectual content. FB, DW, and KM finally approved the version to be published and agreed to be accountable for all aspects of the work in ensuring that questions related to the accuracy or integrity of any part of the work are appropriately investigated and resolved.

## Conflict of Interest Statement

The authors declare that the research was conducted in the absence of any commercial or financial relationships that could be construed as a potential conflict of interest.
